# The Symptoms and Signs of Duodenal Ulcer
*Communicated to the Bristol Medico-Chirurgical Society, November 14th, 1928.


**Published:** 1928

**Authors:** T. Carwardine

**Affiliations:** Consulting Surgeon, Bristol Royal Infirmary


### THE SYMPTOMS AND SIGNS OF DUODENAL ULCER.*

BY

T. Carwardine, M.S.,
*Consulting Surgeon, Bristol Royal Infirmary.*

---

As the diagnosis of duodenal ulcer presents many difficulties, I have made a careful analysis from my personal notes of the symptoms described by 62 patients who had definite duodenal ulcers.

There were 47 males and 15 females, a proportion of three males to one female. The average age was 48·3 years, the youngest being 26 and the oldest 74. In more than half the age lay between 40 and 60. In 12 per cent. the symptoms began by the time the patients came of age ; one each at 15, 16 and 17, three at 18, one at 19, and one each at 21, 23 and 25. The sex incidence was about the same as in later life.

In 12 cases, or 20 per cent., the duration of symptoms was described as " years," prior to surgical treatment ; in 43 cases the period averaged 13 years, including 5 under a year, before coming under surgical care.

The majority used the following terms to describe their sensations : flatulence, dull aching, indigestion, gnawing, pain. Others used the following expressions : discomfort, severe—doubling up, sharp—like a knife, inconvenience, continuous, a very full feeling, a dull

* Communicated to the Bristol Medico-Chirurgical Society, November 14th, 1928.

heavy feeling, raw feeling—smarting, vague flatulence, slight at first—later acute.

The site of these sensations was epigastric, or epigastric and towards the right hypochondrium; "under the breast bone," or "under the right ribs;" occasionally through to the back on the right side, but not round the waist—and rarely felt on the left side.

The relation to food is an important diagnostic point. The average time of onset is one and a half hours after meals. It is generally between one and two hours after meals, but the onset may be as soon as fifteen minutes after food, or as late as from three to seven hours. It may last a short time, or till the next meal is taken, when relief comes for a time, with, in some cases, an increase of the symptoms afterwards. In different patients it may have no relation to food, be relieved by food, or (occasionally) be increased by it.

The symptoms are frequently worse towards evening, and undue importance has been attached to nocturnal pain as a diagnostic point. It was worse by day in 21 patients, by night in 17. When by night the time varies from 11.0 p.m. to 3.0 a.m. in different patients. A few have been unable to sleep unless their stomachs were emptied, by induced vomiting or by washing out.

The symptoms may recur several times in a week, or be absent for as long as six months, only to recur in winter, or in spring and autumn. They tend to be worse in cold weather, and occasionally it is definitely stated that worry increases them. Milk, soda, etc., relieve in most cases—in over one-third of the patients the relief has been very definite. Occasionally, but rarely, alkalis do not relieve the symptoms.

Tenderness is not known to the patients as a rule, but can be elicited by the medical observer in about two-thirds by deep pressure during inspiration. The site is deeper and rather nearer the middle line than that associated with gall-bladder trouble.

Sometimes no loss of weight is apparent, but generally there is some reduction, frequently between one and two stone. Wasting is most marked when patients abstain from food or when the stomach is very dilated.

Hæmatemesis, or melæna, or both, are recorded in less than 20 per cent., and mostly in cases diagnosed some years ago. They are mentioned less commonly now that duodenal ulcer is more frequently diagnosed in the absence of hemorrhage, and the present-day percentage of hemorrhage would be about 10 per cent. Anæmia is exceptional, only consequent upon any severe bleeding, and is recovered from very quickly after bleeding has ceased.

Vomiting occurs at some time or other in two-thirds of the cases. It may be slight and occasional, or severe "especially when the attacks are on," and emesis brings relief. It is more common towards the night, and may be self-induced in order to obtain better rest. It occurs chiefly when the attacks are severe, when there is stenosis of the duodenum and consequent gastric dilatation, and when inflammatory swelling, "duodenitis," is present. Gastrectasis is obvious in about a third, and the patient may have resorted to lavage.

Flatulence is usually present. It is one of the most constant symptoms. It may be "slight" or "severe," and when wind is brought up the patient often experiences relief. It may be so severe as to cause a "crowing" sound, and has led to the constant belching of wind in the consulting-room. It may be such a prominent feature as to be mistaken for hysterical flatulence.

Acidity ("water-brash") is generally present when the attacks are on, and may be of various degrees of severity. About a third of the patients give a history of heart-burn. Salivation is sometimes noticed during the attacks, but a few patients complained of dryness of the mouth. One lady—who salivated at times, and whose mouth was dry at other times—was very delighted after operation to find that her saliva had returned and that she was thus able to eat dry foods like bread and toast.

Jaundice is absent in any great degree, but a slight tinge of sclerotics, far back, is by no means uncommon. Constipation is usual during the attacks.

Apart from "periodicity" some patients refer to stages in the symptoms, of which the following are examples, all in men:—

Mr. F., 38. *First stage.*—Before the war, "indigestion." *Second stage.*—During active service, "free." *Third stage.*—After the war, recurrence of symptoms.

Mr. J., 39. *First stage.*—For two or three years a very full feeling an hour after meals, lasting two hours, relieved by sod. bicarb. *Second stage.*—For ten years pain of gnawing character three or four hours after meals, worse at night, relieved by food. *Third stage.*—For five years similar pains, but when he broke wind it tasted like a bad egg. Attacks followed by vomiting and diarrhœa.

Mr. N., 38. When he left school at 14 or 15 he had pains and used to buy biscuits or "anything" to relieve the pain, which was severe, lasted a day or two, and was worse in cold weather. At 38 he abstains from food to relieve his pain, which is now more severe, and he says, " Since vomiting has become frequent the less food I take the less pain I get, but worry makes it worse."

*Summary of Symptoms.*—The patient is usually a male over 40 years of age, and has for many years suffered from periodical flatulence and epigastric discomfort between one and two hours after meals, either by day or night, generally towards evening, and usually relieved by food and alkalis. The patient may vomit with the attack and may complain of heartburn. Hunger pain is not always present, and the symptoms may change with time. The symptoms are liable to be mistaken for those of gall-bladder trouble in particular, but may be simulated by those of appendix dyspepsia and various intra-abdominal adhesions. When a diagnosis of gall-bladder disease is made—unless that diagnosis is very definite—one should always remember the possibility of a duodenal ulcer being present with the risks of a perforation at any time. Very careful radiological examination is imperative and may be conclusive.

*Differential diagnosis.*—This has to be considered in both its medical and surgical aspects. Dr. Robert Hutchison has defined dyspepsia as " any discomfort felt during digestion and due to organic disease of the stomach (or duodenum) or to primary disorder of its functions." There are numerous medical conditions, as well as surgical, which give rise to the deceptive symptoms of vomiting, pain and flatulence. With duodenal ulcer flatulence is a common and almost constant symptom ; distinct pain is exceptional—the patient usually complains of epigastric discomfort of some sort; and vomiting occurs with the attacks, or when stenosis leads to easily recognisable gastric dilatation.

On the surgical side the differential diagnosis is illustrated best by mistakes which have been made, grouped as follows:—

(1) *Diagnosed as gall-bladder disease, but duodenal ulcer present.*

This group comprises six cases with symptoms of ten to twenty years' duration. In one case the pain was relieved by food, in another made worse thereby. In three there was back pain below the right scapula. In two the pain was worse at night. Flatulence and sickness were symptoms. In one there was jaundice accompanied by pyrexia and shivering with the attacks, and Boas' hyperæsthesia.

One lady, whom I diagnosed as having gall-stones, had a perforation of her duodenal ulcer some months after, and her doctor's early recognition of this led to her recovery.

(2) *Diagnosed as possible gall-bladder disease or duodenal ulcer.*

In cases under this heading flatulence and sickness were present, and there was a slight tinge of the sclerotics. The X-ray diagnosis of duodenal ulcer proved the correct one.

(3) *Combined duodenal ulcer and gall-bladder disease.*

There were two of these cases; in both there were duodenal ulcers with cholecystitis—one with stones—and in both the gall-bladder was removed and an anastomosis done.

(4) *Diagnosed as duodenal ulcer, but gall-bladder trouble present without ulcer.*

Of this again there were two instances. The symptoms were not definite enough for diagnosis. One patient, with a kinked and adherent cystic duct, was only relieved by chewing gum.

(5) *Bands with symptoms like those of duodenal ulcer.*

There were four of these cases, one with fulness after food, two with hunger pain relieved by food, and one had abnormal appetite. The bands were in the following positions: one, with a typical text-book history of duodenal ulcer, but no ulcer, had a strong band under the transverse colon. One had a band to the small intestine with visible pyloric spasm, another had matted small intestine, the fourth had various bands and a calcareous gland in the cæcal region.

(6) *Appendix dyspepsia.*

Of this there were two probable examples. One had "terrible hunger" relieved by food and alkalis, and with pain worse at midnight. She had been eight weeks on Lenhartz treatment, and showed great and rapidly changing spasm of the pyloric antrum and of the duodenum at the time of operation.

The remainder comprise two cases which turned out to be malignant pancreatic disease, and one of cancerous ulcer of the duodenum. Another, a duodenal ulcer, owing to the inflammatory tumour was diagnosed as cancer of the hepatic flexure. One (not operated upon) was probably an example of diverticulosis of the stomach.

A post-operative review of the symptoms in these cases affords little ground for thinking that the clinical diagnosis might have been more exact, although full advantage of X-ray examination should be taken. Moreover, diagnosis is rendered difficult when adhesions to other organs are present. Adhesions to the following parts were found: to the gall-bladder, thirteen cases, including three which were adherent to the liver also, and one to the omentum in addition; to the liver, seven cases, in three adherent to the gall-bladder also, and in one to the stomach in addition; and to the pancreas in six or seven cases. Occasionally adhesions are met with to the abdominal wall, or extensive adhesions elsewhere. Many of the difficulties in diagnosis may be explained by the anatomical proximity of the duodenum and gall-bladder, and the frequency of pathological adhesions, particularly to the gall-bladder.

Malignancy is rarely associated with duodenal ulcer; but when an ulcer burrows into the pancreas, eroding it and forming a distinct crater, there is in my opinion a strong suspicion of malignant change, with a tendency to serious and fatal hemorrhage. The following case is particularly interesting:—

In 1901 the late Dr. Herschell saw with me a gentleman, aged 72. He found an absence of free acid, demonstrated the presence of the Oppler-Boas bacillus, and therefore diagnosed a gastric ulcer becoming malignant. The condition turned out to be a duodenal ulcer, and the usual anastomosis was done. But twelve years after, at the age of 84, the patient died of cancer of the stomach.

The possibility of the symptoms being reflex must be thought of. A nurse had indigestion for some years, pain two hours after meals and lasting till the next meal. She had much flatulence, and was rigid and tender in the right hypochondrium. Operation was proposed, but a rectal examination detected a retroverted uterus, the replacement of which effected a cure.

Accidents may happen. Whilst awaiting operation, one patient died of hemorrhage from duodenal ulcer, and two have perforated.

*X-ray diagnosis.*—During the last few years very rapid strides have been made by radiologists in the detection and portraiture of duodenal ulcers. As with gastric ulcer, so with duodenal ulcer, the signs must now be regarded as radiological. With suitable apparatus, skill and perseverance, the ulcer may be shown on the X-ray film as an islet, or a niche with a notch opposite, which are quite diagnostic—in my experience more definite and certain even than the casual and necessarily imperfect inspection of the duodenum at operation. Indications of this progress were given in a previous paper,[1] with examples by Dr. Mayes of the actual photograph of the ulcer, with its central spot, perhaps a mottled halo, and a shadow around—seen sideways or face-view ; and since then other cases have occurred in which the diagnosis has been made, or the clinical diagnosis corrected, before operation, by the photography of the ulcer.

Whether medical or surgical treatment be adopted, or medical measures be employed throughout, the diagnosis is all-important ; and it can, and can only, be made accurately by competent X-ray examination, preferably by taking a positive picture from the X-ray negative. I would urge medical men to make the fullest possible use of this diagnostic procedure, and not to rely on history and clinical signs alone, or for want of its use to miss a duodenal ulcer which may perforate at any time. Moreover, it is possible that improvements in technique may soon afford radiologists the opportunity of verifying the results of treatment from time to time. This would be the best proof of the efficacy or otherwise of any medical measures adopted.

### REFERENCE.

[1] *Bristol Medico-Chirurgical Journal,* 1924, xli., 16.

